# Tirzepatide for the Maintenance of Body Weight Reduction: Rationale, Design, and Baseline Characteristics of SURMOUNT‐MAINTAIN

**DOI:** 10.1002/oby.70014

**Published:** 2025-09-07

**Authors:** Deborah B. Horn, Louis J. Aronne, Sean Wharton, Harold E. Bays, Elisa Gomez‐Valderas, Avigdor D. Arad, Palash Sharma, Julia P. Dunn, Cagri Senyucel, Clare J. Lee

**Affiliations:** ^1^ University of Texas Center for Obesity Medicine and Metabolic Performance, Department of Surgery University of Texas McGovern Medical School Houston Texas USA; ^2^ Comprehensive Weight Control Center, Division of Endocrinology, Diabetes & Metabolism, Weill Cornell Medicine New York New York USA; ^3^ University of Toronto and Wharton Weight Management Clinic Toronto Ontario Canada; ^4^ Louisville Metabolic and Atherosclerosis Research Center Louisville Kentucky USA; ^5^ Eli Lilly and Company Indianapolis Indiana USA

**Keywords:** antiobesity medication, obesity, obesity management medication, SURMOUNT‐MAINTAIN, tirzepatide

## Abstract

**Objective:**

SURMOUNT‐MAINTAIN aims to evaluate the efficacy and safety of reducing the tirzepatide dose and/or continuing the maximum tolerated dose (MTD) versus placebo in maintaining body weight (BW) reduction achieved with tirzepatide MTD.

**Methods:**

This Phase 3b, multicenter, randomized, parallel‐arm, double‐blinded, placebo‐controlled, 52‐week clinical trial is in progress comparing treatment with once weekly tirzepatide (5 mg and/or MTD of 15 mg or 10 mg) versus placebo in achieving BW reduction maintenance from the initial 60‐week open‐label weight‐loss period on tirzepatide MTD, in adults with obesity (BMI ≥ 30 kg/m^2^ or ≥ 27 kg/m^2^ with ≥ 1 obesity‐related comorbidity, excluding type 2 diabetes). The primary endpoint is percent maintenance of BW reduction achieved during the weight‐loss period at Week 112 among those who reached a BW plateau (i.e., < 5% BW change) between Weeks 48 and 60.

**Results:**

Participants are mostly female (65%) with a mean ± SD age of 47 ± 13 years, BW 114 ± 27 kg, BMI 40 ± 8 kg/m^2^, and waist circumference 119 ± 18 cm.

**Conclusions:**

The SURMOUNT‐MAINTAIN trial will evaluate whether reducing or continuing the tirzepatide dose as a long‐term treatment option may help maintain the reduced BW initially achieved with tirzepatide MTD versus switching to placebo. Combined, this study may provide additional evidence to help tailor patient‐centered strategies for maintenance of BW reduction in adults living with obesity.

**Trial Registration:**

ClinicalTrials.gov identifier: NCT06047548

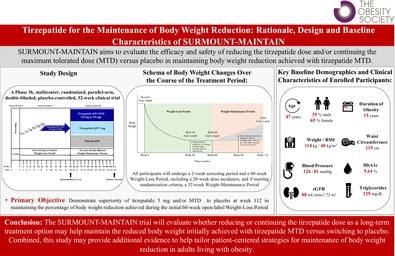

## Graphical abstract

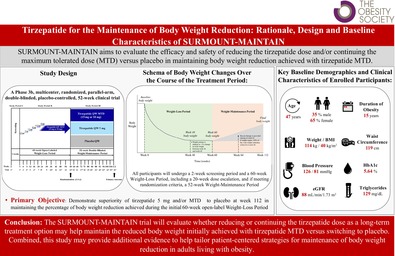

**Study Importance**

- What is already known?
  ○ Obesity is a chronic disease requiring long‐term treatment. Discontinuation of obesity management medication leads to weight regain despite continued lifestyle intervention.
  ○ Tirzepatide is a once weekly glucose‐dependent insulinotropic polypeptide (GIP) and glucagon‐like peptide‐1 (GLP‐1) receptor agonist approved for the treatment of type 2 diabetes and obesity.
  ○ In the SURMOUNT clinical trial program, robust weight reduction was observed with tirzepatide in people with obesity or overweight, with and without type 2 diabetes, compared to placebo.
- What does this study add?
  ○ The SURMOUNT‐MAINTAIN clinical trial is designed to evaluate whether participants with reduced body weight after treatment with the maximum tolerated dose of tirzepatide are able to maintain weight reduction by: (1) reducing the dose of tirzepatide or (2) continuing the maximum tolerated dose and to compare each strategy to switching to placebo.
  ○ This study will also generate data on clinically relevant concepts such as “body weight plateau” defined in this trial as less than 5% body weight change within 12 weeks following an initial 60‐week weight‐loss period; and “medication rescue” with obesity management medication, in this trial tirzepatide, for those who regained half or more of body weight reduction achieved during the weight‐loss period.
- How might these results change the direction of research or the focus of clinical practice?
  ○ Results from the SURMOUNT‐MAINTAIN trial (anticipated completion date May 2026) will provide data on the efficacy and safety of reducing the weekly dose of tirzepatide from the maximum tolerated dose to 5 mg and/or continuing the maximum tolerated dose versus placebo for the maintenance of body weight reduction.
  ○ Data generated from this study is intended to inform and guide clinicians and patients on long‐term options with tirzepatide treatment for body weight reduction maintenance.

## 1 Introduction

Obesity is a chronic disease, and its increasing prevalence is a public health concern given the associated risk of comorbidities, including cardiovascular disease, and many additional cardiometabolic diseases such as type 2 diabetes (T2D), hypertension, dyslipidemia, sleep apnea, and hypertension, as well as cancer and premature death [1, 2, 3, 4, 5]. The risk of obesity‐related comorbidities is reduced by sustained body weight reduction [6].

Treatment with obesity management medications (OMMs) as adjunct to lifestyle intervention leads to a greater and more sustained body weight reduction compared to lifestyle intervention alone [7, 8, 9, 10]. Although it is well established that obesity treatment improves morbidity and mortality [11, 12, 13], the cardiometabolic risk factor benefits achieved with body weight reduction during OMM treatment have been shown to deteriorate with the rapid weight regain, also known as weight recurrence, that occurs from OMM discontinuation [9, 10, 14]. In SURMOUNT‐4, the 36‐week lead‐in period with tirzepatide treatment resulted in a mean 21% body weight reduction, which was followed by substantial (14%) weight regain in participants randomized to switch to placebo [15]. Similarly, in the STEP 4 trial following a 20‐week run‐in period of semaglutide treatment [10], participants also regained substantial (7%) body weight when switched to placebo. This effect was further supported by the STEP 1 extension trial, wherein 1 year after discontinuation of semaglutide and the lifestyle intervention program, participants had regained two‐thirds of their prior weight reduction and partial reversal of improvements in cardiometabolic metrics [14]. The evidence supports that maintenance of health benefits of weight reduction from OMMs is best achieved when OMM treatment is maintained [16].

Tirzepatide is a once weekly, single molecule dual glucose‐dependent insulinotropic polypeptide (GIP) and glucagon‐like peptide‐1 (GLP‐1) receptor agonist approved in adults for the treatment of T2D, obesity, and (in the US) moderate to severe obstructive sleep apnea in patients with obesity. In the global Phase 3 SURMOUNT clinical trial program, for people with obesity or overweight, with and without T2D, tirzepatide demonstrated clinically significant and clinically meaningful body weight reduction and improvement in markers of cardiometabolic comorbidities [15, 17, 18, 19]. In SURMOUNT‐1, participants with overweight and obesity demonstrated mean body weight reduction ranging from 16% to 23% after Week 72 with tirzepatide treatment [17]. The results suggest that clinically meaningful improvements in body weight reduction are anticipated for those randomized to and remaining on tirzepatide. However, it is not known if people receiving tirzepatide treatment for weight management can maintain their body weight reduction with a reduced dose of tirzepatide. Similarly, data are limited on the effect of long‐term treatment with the maximum tolerated dose (MTD) of tirzepatide on the maintenance of body weight reduction. Herein is a presentation of the clinical trial design, objectives, endpoints, and baseline characteristics of SURMOUNT‐MAINTAIN, the first clinical trial assessing the effect of tirzepatide, either at a reduced dose and/or at the continued MTD compared to switching to placebo, on the maintenance of body weight reduction achieved with the MTD of tirzepatide (15 mg or 10 mg).

## 2 Methods

### 2.1 Study Design

**FIGURE 1:**
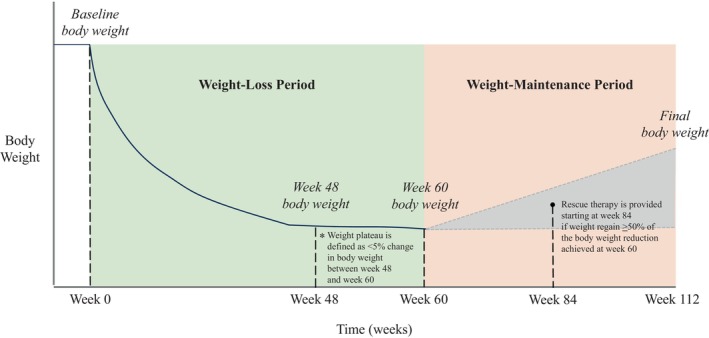
Maintenance of body weight reduction Schema of body weight changes over the course of the treatment period. Definitions of key events including weight‐loss period, weight plateau, and weight‐maintenance period are provided in Table 1. [Color figure can be viewed at wileyonlinelibrary.com]

SURMOUNT‐MAINTAIN is a Phase 3b, multicenter, randomized, parallel‐arm, double‐blinded, placebo‐controlled, 52‐week clinical trial comparing weekly tirzepatide 5 mg and/or MTD (15 mg or 10 mg) versus placebo in achieving maintenance of body weight reduction from the initial 60‐week open‐label weight‐loss period on tirzepatide MTD, in participants who have obesity or overweight with at least one obesity‐related comorbidity but without T2D (Figure 1). Participating sites included US academic medical centers, private research sites, specialty practices, and primary care practices. After completing the 60 weeks of the weight‐loss period, participants meeting randomization criteria will be randomly assigned in a 3:3:2 ratio to (1) continue treatment with tirzepatide MTD, (2) reduce the dose to 5 mg, or (3) switch to placebo. Eligibility criteria for randomization will include (1) tolerating tirzepatide 10 mg or 15 mg dose without a change in tirzepatide dose between Week 48 and Week 60 (randomization visit), and (2) achieving body weight reduction of at least 5% at Week 60 from Week 0. For participants who meet randomization eligibility criteria, stratification factors will be assessed, which are (1) Yes/No, weight reduction of at least 20% at Week 60 from Week 0 was achieved, (2) Yes/No, body weight plateau was reached at Week 60, and (3) sex (female or male). Body weight plateau will be defined as less than 5% body weight change between Week 48 and Week 60. Assignment to treatment groups will be determined by a computer‐generated random sequence using an interactive web‐response system.

**FIGURE 2:**
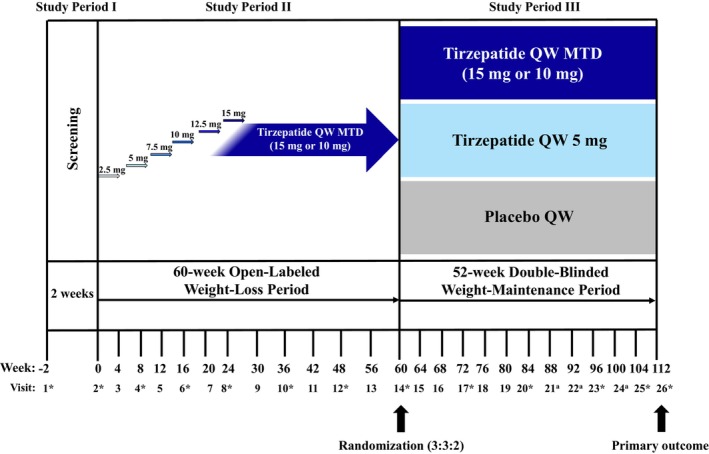
Study design This is a Phase 3b, multicenter, randomized, parallel‐arm, double‐blinded, placebo‐controlled, 52‐week clinical trial comparing the efficacy and safety of tirzepatide 5 mg QW and/or tirzepatide MTD (15 mg or 10 mg) QW versus placebo in achieving maintenance of body weight reduction from the initial 60‐week open‐label weight‐loss period on tirzepatide MTD, in participants who have obesity or overweight with at least 1 weight‐related comorbidity but without type 2 diabetes. *In‐clinic body weight measurement is to be taken during the visit. Body weight must be measured in the fasting state, except for Visits 6 and 10. If the participant is not fasting, the participant should return at a later date within the visit interval tolerance in the fasting state. Visits without an asterisk are telehealth visits, which may be converted to in‐clinic visits if clinically indicated. ^a^Body weight measurement is applicable only if telehealth Visit 21, Visit 22, or Visit 24 is converted to an in‐clinic visit to assess eligibility for rescue tirzepatide. MTD, maximum tolerated dose; QW, once weekly. [Color figure can be viewed at wileyonlinelibrary.com]

All participants will undergo a 2‐week screening period and a 60‐week weight‐loss period, including a 20‐week dose escalation, and, if meeting randomization criteria, a 52‐week weight‐maintenance period (Figure 2). All study participants will receive lifestyle intervention throughout the trial, including dietary counseling by a dietitian or nutritionist, or equivalent qualified delegate, according to local standard. Dietary counseling consists of advice on healthy food choices and focus on calorie restriction using a hypocaloric diet with macronutrient composition of maximum 30% of energy from fat, approximately 20% of energy from protein, approximately 50% of energy from carbohydrates, and an energy deficit of approximately 500 kcal/day compared to the participant's estimated total energy expenditure. Additionally, participants will be advised to aim to increase their physical activity to at least 150 min/week. A participant's nutritional needs and hydration status will be monitored by medical staff (via history and physical and laboratory assessment as needed) if there is a report of significantly reduced caloric intake. If BMI ≤ 22 kg/m^2^ is reached in participants receiving study intervention, the recommended energy intake should be recalculated with no kcal deficit for the remainder of the trial. A full list of key term definitions used in this study is provided in Table 1.

**TABLE 1:** Key definitions.

Key term	Study definition
Weight‐loss period	The first 60 weeks of tirzepatide treatment provided to all qualifying participants in an open‐label manner
Weight plateau	< 5% BW change over 3 months (between Week 48 and Week 60) after intentional BW reduction (i.e., with tirzepatide treatment) 100 × (Week 60 BW − Week 48 BW)/Week 48 BW
Weight‐maintenance period	The 52 weeks following weight‐loss period during which time qualifying participants are randomized 3:3:2 to continue tirzepatide MTD or reduce the dose to 5 mg or to switch to placebo in a double‐blinded manner
Weight regain	100 × (Current BW − Week 60 BW)/(Baseline BW – Week 60 BW)
Rescue treatment	For weight regain of 50% or more based on above weight regain formula, rescue tirzepatide will be provided to the study participant to limit the maximum weight regain possible in the study and to limit the potential metabolic harm associated with weight regain
Maintenance of weight reduction	
Percent maintenance of BW reduction achieved during the 60‐week weight‐loss period	100 × (Final BW − Baseline BW)/(Week 60 BW − Baseline BW)
Maintaining ≥ 80% of the BW reduction achieved during the 60‐week weight‐loss period	Those with at least 80% using above formula are considered maintainers
Maintaining ≥ 15% BW reduction for participants who have already lost ≥ 15% BW at randomization (Week 60)	Among those who have achieved ≥ 15% BW reduction at Week 60, how many participants also meet ≥ 15% BW reduction at Week 112 from baseline?

*Note*: Current BW refers to the body weight measured in clinic for weight regain evaluation.

Abbreviations: BW, body weight; MTD, maximum tolerated dose.

### 2.2 Study Participants

Key inclusion criteria will include adults ≥ 18 years with BMI ≥ 30 kg/m^2^ or ≥ 27 kg/m^2^ with at least one weight‐related comorbidity (e.g., hypertension, dyslipidemia, obstructive sleep apnea, cardiovascular disease) and a history of at least one self‐reported unsuccessful dietary effort to lose body weight (Table 2). Key exclusion criteria will include a previous diagnosis of diabetes or at least one laboratory value suggestive of diabetes during screening, self‐reported change in body weight > 5 kg within 3 months prior to screening, and any use of GLP‐1 receptor agonist or GIP/GLP‐1 receptor agonist within 12 months of screening (Table 2). A complete list of eligibility criteria is available in the online Supporting Information Appendix S1. Female enrollment will be capped at 70% to ensure a sufficiently large sample of males. This trial will be conducted in accordance with good clinical practice guidelines and the principles of the Declaration of Helsinki [20]. Independent Ethics Committee or Institutional Review Board approval was received for each of the participating sites. All participants provided written informed consent prior to trial participation.

**TABLE 2:** Key eligibility criteria.

**Key inclusion criteria**
Adult male or female ≥ 18 years^a^
BMI ≥ 30 kg/m^2^ or
BMI ≥ 27 kg/m^2^ with ≥ 1 previously diagnosed weight‐related comorbidity: hypertension, dyslipidemia, obstructive sleep apnea, cardiovascular disease
History of ≥ 1 self‐reported unsuccessful dietary effort to lose body weight
**Key exclusion criteria**
Diabetes‐related
History of T1D
History of T2D
History of ketoacidosis, hyperosmolar state, or coma
At least one laboratory value suggestive of diabetes during screening, including one or more of HbA1c ≥ 6.5%, fasting glucose ≥ 126 mg/dL, or random glucose ≥ 200 mg/dL
Obesity‐related
Self‐reported change in body weight > 5 kg within 3 months prior to screening
Prior or planned surgical treatment for obesity, excluding liposuction or abdominoplasty if performed > 1 year prior to screening
Prior or planned endoscopic and/or device‐based therapy for obesity or device removal within the last 6 months prior to screening (e.g., mucosal ablation, gastric artery embolization, intragastric balloon, duodenal‐jejunal endoluminal liner)
Other medical
Renal impairment eGFR < 30 mL/min/1.73 m^2^
History of clinically significant gastric emptying abnormality (e.g., severe gastroparesis, gastric outlet obstruction) or chronic use of medication that directly affects gastrointestinal motility
History of chronic or acute pancreatitis
Uncontrolled endocrine abnormality in the opinion of the investigator (e.g., thyrotoxicosis, adrenal crises)^b^
Obesity induced by other endocrinologic disorders (e.g., Cushing syndrome) or diagnosed monogenetic or syndromic forms of obesity (e.g., melanocortin 4 receptor deficiency, Prader Willi syndrome)
History of significant active or unstable major depressive disorder or other severe psychiatric disorder (e.g., schizophrenia, bipolar disorder, other serious mood or anxiety disorder) within the last 2 years^c^
Uncontrolled hypertension (systolic blood pressure ≥ 160 mmHg and/or diastolic blood pressure ≥ 100 mmHg)
Acute myocardial infarction, cerebrovascular accident (stroke), unstable angina, or hospitalization due to congestive heart failure within 3 months prior to Visit 2
NYHA functional classification IV congestive heart failure
Acute or chronic hepatitis or other liver disease (excluding nonalcoholic fatty liver disease^d^) or ALT level > 3× ULN for the reference range, ALP level > 1.5× ULN, or total bilirubin > 1.5× ULN, except for Gilbert's syndrome
Serum calcitonin level ≥ 20 ng/L, if eGFR ≥ 60 mL/min/1.73 m^2^ and ≥ 35 ng/L, if eGFR < 60 mL/min/1.73 m^2^, at Visit 1
Family or personal history of MTC or MEN syndrome type 2
History of active or untreated malignancy or remission from clinically significant malignancy (other than basal or squamous cell skin cancer, in situ carcinomas of the cervix, or in situ prostate cancer) for < 5 years
Any other condition not listed (e.g., hypersensitivity, intolerance) that is a contraindication to GLP‐1 receptor agonists or GIP/GLP‐1 receptor agonists
History of any other condition that, in the opinion of the investigator, may preclude the participant from following and completing the protocol (e.g., known drug or alcohol abuse, diagnosed eating disorder, other psychiatric disorder)
History of marijuana use or tetrahydrocannabinol‐containing products within 3 months of enrollment or unwillingness to abstain from marijuana or tetrahydrocannabinol‐containing products use during the trial^e^
Transplanted organ (corneal transplants [keratoplasty] allowed) or awaiting an organ transplant
Any hematological condition that may interfere with HbA1c measurement (e.g., hemolytic anemias, sickle cell disease)
Prior/concomitant therapy
Use of GLP‐1 receptor agonists (prescribed or in a clinical study) within 12 months of screening
Receiving or have received within 3 months prior to screening chronic systemic glucocorticoid therapy (excluding topical, intraocular, intranasal, intra‐articular, or inhaled preparations)
Medications that may cause weight gain (e.g., tricyclic antidepressants, atypical antipsychotics, mood stabilizers)^f^
Medication or alternative therapies that promote weight loss

Abbreviations: ALP, alkaline phosphatase; ALT, alanine aminotransferase; eGFR, estimated glomerular filtration rate; GIP, glucose‐dependent insulinotropic polypeptide; GLP‐1, glucagon‐like peptide‐1; MEN, multiple endocrine neoplasia; MTC, medullary thyroid cancer; NYHA, New York Heart Association; T1D, type 1 diabetes; T2D, type 2 diabetes; ULN, upper limit of normal.

^a^ Male participants with partners of childbearing potential should be willing to use reliable contraceptive methods throughout the study and for 5 half‐lives of the study drug plus 90 days; female participants of childbearing potential require negative screening for pregnancy before randomization, and contraception counseling is provided to avoid pregnancy during the trial and soon after the trial is completed; female participants of childbearing potential who are sexually active must agree to use two forms of effective contraception, where at least one form is highly effective for the duration of the trial plus 30 days (females who had bilateral tubal ligation should use second form of contraception).

^b^ Participants receiving treatment for hypothyroidism may be included, provided their thyroid hormone replacement dose has been stable for at least 3 months. Participants with a history of subclinical hypothyroidism may be included if, in the investigator's opinion, the participant is unlikely to require initiation of thyroid hormone replacement during the course of the study.

^c^ Participants with major depressive disorder or generalized anxiety disorder whose disease state is considered stable for the past 2 years and expected to remain stable throughout the course of the study, in the opinion of the investigator, may be considered for inclusion if they are not on excluded medications.

^d^ Participants with nonalcoholic fatty liver disease are eligible to participate in this trial if their ALT level is ≤ 3.0× ULN for the reference range.

^e^ If a participant has used cannabidiol oil during the past 3 months but agrees to refrain from use for the duration of the study, the participant can be enrolled.

^f^ Selective serotonin reuptake inhibitors other than paroxetine are permitted.

### 2.3 Dose Escalation and Modification

All enrolled participants initiate treatment with tirzepatide 2.5 mg and increase by 2.5 mg every 4 weeks. The protocol will provide guidance to assist sites and participants to escalate participants to the 15 mg dose or MTD (15 mg or 10 mg) during the initial dose escalation. Doses and associated escalation schemes were based on data from Phase 1, 2, and 3 clinical trials in participants with T2D [21, 22, 23, 24, 25, 26, 27]. Tirzepatide doses of 5 mg or MTD (15 mg or 10 mg) have been selected for the weight‐maintenance period based on the following criteria: (1) each dose provides clinically meaningful weight reduction relative to placebo, and (2) safety and tolerability are supported by results and data from SURMOUNT‐1 [17].

Dose modification will be permitted for management of intolerable gastrointestinal symptoms or in case of a participant reaching BMI ≤ 22 kg/m^2^ while on tirzepatide. Only one cycle of dose de‐escalation and re‐escalation in response to persistent intolerability will be permitted during the open‐label weight‐loss period. Participants who do not tolerate at least 10 mg by Visit 12 (Week 48) will not be eligible for randomization and will be discontinued from the study and from the study drug. Dose de‐escalation and re‐escalation will not be allowed during the weight‐maintenance period.

### 2.4 Rescue Treatment

Starting at Week 84, if a participant reaches or exceeds the 50% weight regain from body weight reduction achieved at Week 60, the participant will receive rescue treatment with re‐initiation of tirzepatide or increase in the dose of tirzepatide depending on the randomization assignment while maintaining the blinding (Table 1). The body weight measurements used to determine rescue treatment eligibility must be obtained from in‐clinic fasting visit measurements.

### 2.5 Discontinuation

If study intervention is discontinued during the open‐label weight‐loss period, the participant will not be randomized and will be discontinued from the study. If study intervention is discontinued after randomization, the participant will be encouraged to continue to attend all scheduled study visits and collect all planned efficacy and safety measurements.

Clinical considerations for discontinuation of study drug will include initiation of GLP‐1 receptor agonist or dipeptidyl peptidase‐4 (DPP‐4) inhibitor, GIP/GLP‐1 receptor agonist obtained outside the study, or initiation of additional FDA‐approved prescription OMMs if participants cannot discontinue them. Additional considerations include if the participant has bariatric surgery or a weight‐loss procedure, becomes pregnant, or has significantly elevated calcitonin levels or if the investigator believes that study intervention discontinuation is appropriate due to the occurrence of any other treatment‐emergent adverse event (TEAE), serious adverse event (SAE), or clinically significant finding. If BMI ≤ 18.5 kg/m^2^ is reached at any time during the treatment period, or the participant has significant gastrointestinal symptoms despite management, the investigator will be advised to contact the sponsor medical monitor to discuss whether continuing the study treatment is medically appropriate. A full list of clinical considerations for study discontinuation is provided in the online Supporting Information Appendix S1. A participant will be considered lost to follow‐up if they repeatedly fail to return for scheduled visits and cannot be contacted by the study site (Appendix S1).

### 2.6 Objectives and Outcome Measures

The primary objective of this study is to demonstrate that tirzepatide 5 mg and/or MTD are superior to placebo at Week 112 in maintaining the percentage of body weight reduction achieved during the initial 60‐week open‐label weight‐loss period. Eligibility for this analysis is restricted to participants who have achieved at least a 5% reduction in body weight and reached a plateau by the end of the 60‐week period, between Week 48 and Week 60 as defined in Table 1. The primary endpoint is defined as the percentage of maintenance of body weight reduction, ranging from 0% to 100%, and the formula is provided in Table 1. Of note, a percentage maintenance of 0% indicates a complete regain of lost weight by Week 112, suggesting that the participant's weight at the end of the study equals or exceeds the baseline weight. In contrast, a percentage maintenance of 100% or above indicates that the participant's weight at Week 112 is equal to or lower than the weight at Week 60, signifying full maintenance of the weight loss achieved during the open‐label weight‐loss period.

Key secondary objectives will be to demonstrate the superiority of tirzepatide 5 mg and/or MTD compared to placebo in: (1) maintaining ≥ 80% of the body weight reduction achieved during the 60‐week weight‐loss period and (2) maintaining ≥ 15% body weight reduction for participants who have already lost ≥ 15% body weight at randomization (Week 60) including only participants who reached the body weight plateau by the end of the weight‐loss period (Week 60). In addition, we plan to evaluate the superiority of tirzepatide 5 mg and/or continuing the MTD compared to switching to placebo in percent change in body weight at trial end (Week 112) from Week 0 and include all participants regardless of whether they have reached the body weight plateau. The aim of each estimand is described in the online Supporting Information Appendix S1.

### 2.7 Assessments

Efficacy assessments of all randomized participants will be collected throughout the trial as specified in the protocol and include fasting body weight, BMI, waist circumference, fasting insulin, HbA1c, fasting plasma glucose, blood pressure, and fasting lipids. A central laboratory will be used for all laboratory assessments. Body composition will be assessed by dual‐energy x‐ray absorptiometry (DXA) in a subset of study participants measured at baseline, Week 60, prior to starting rescue tirzepatide, and early termination or Week 112, with the intent of evaluating changes in body composition including total fat mass and total lean mass associated with body weight reduction and maintenance. This subset will include the first 200 participants who were enrolled and consented to both the main study and the DXA addendum.

Safety assessments will be carried out throughout the trial as specified in the protocol and include a complete physical examination (including cardiovascular, respiratory, gastrointestinal, and neurological systems and thyroid examination), blood pressure, pulse, electrocardiograms (ECGs), and laboratory assessments (including hepatic, renal, pancreatic, calcitonin, hematology, and immunogenicity assessments). In addition, participants will be monitored for depression, suicidal ideation, and behavior risk through mental health questionnaires (Patient Health Questionnaire‐9 [PHQ‐9] [28] and the Columbia‐Suicide Severity Rating Scale [C‐SSRS] [29, 30]). Adverse event and concomitant medication information will be collected throughout the trial, including safety follow‐up.

### 2.8 Statistical Analyses

Approximately 400 participants will be enrolled in the open‐label weight‐loss period to randomize approximately 320 participants with a 3:3:2 ratio to tirzepatide MTD (15 mg or 10 mg), tirzepatide 5 mg, and placebo arm, respectively. From these randomized participants, it will be expected that at least 240 (90/90/60) will have reached the body weight plateau at Week 60 and will be entered in the primary analysis. This sample size was selected to provide approximately 95% ability to detect a 10% difference in mean percent maintenance at Week 112 of body weight reduction achieved during the 60‐week weight‐loss period between tirzepatide 5 mg versus placebo or tirzepatide MTD versus placebo. The evaluation of the superiority of tirzepatide 5 mg or MTD to placebo will be conducted at an alpha level of 0.025 for each comparison to allow an overall 0.05 alpha level for the study.

Primary and key secondary efficacy endpoints will be analyzed using the modified intent‐to‐treat (mITT) population for efficacy analyses comprised of all randomized participants who were exposed to at least one dose of study drug, excluding those participants who were inadvertently enrolled. The efficacy estimand will be evaluated on the efficacy analysis set (EAS) which is based on the mITT for efficacy analyses population excluding data after treatment discontinuation, initiation of other OMMs, GLP‐1 receptor agonists, GIP/GLP‐1 receptor agonists, DPP‐4 inhibitors, or rescue tirzepatide, or having bariatric surgery or other weight‐loss procedures. The modified treatment‐regimen estimand will be evaluated in the full analysis set (FAS), which is based on the mITT for efficacy analyses population regardless of adherence to study treatment and regardless of initiation of other OMMs, GLP‐1 receptor agonists, GIP/GLP‐1 receptor agonists, or DPP‐4 inhibitors. Data obtained after rescue tirzepatide or having bariatric surgery or other weight‐loss procedures will be excluded.

For the efficacy estimand, missing data at Week 112 for participants who take rescue tirzepatide will be imputed with their highest body weight measurement taken after randomization and prior to starting rescue. No additional imputation will be performed. A mixed model for repeated measures (MMRM) will be used as the primary analysis model for maintenance of body weight reduction over time. For the modified treatment‐regimen estimand, the primary efficacy analysis will be conducted using a covariance (ANCOVA) model to analyze percent maintenance at Week 112 of body weight reduction achieved during the 60‐week weight‐loss period. For the treatment‐regimen estimand, missing body weight values at the 112‐week visit in participants who had bariatric surgery or another weight‐loss procedure or took rescue tirzepatide will be imputed with the highest body weight measurement collected after randomization and before the participants had surgery or took rescue tirzepatide. Missing body weight values at the 112‐week visit in participants who discontinued study intervention early and did not have bariatric surgery or another weight‐loss procedure and did not take rescue tirzepatide will be imputed based on observed data in the same treatment group from participants who had their efficacy assessed after early discontinuation of study intervention after randomization (Week 60). This analysis will be conducted with multiple imputations, and statistical inference over multiple imputations will be guided by the method proposed by Rubin [31].

Key secondary objectives will be controlled for type I error and evaluated based on both estimands. Among the participants who reached the body weight plateau at Week 60, logistic regression will be used to assess participants achieving ≥ 80% maintenance at Week 112 of body weight reduction from the 60‐week weight‐loss period and achieving ≥ 15% body weight reduction at Week 112 for those who have already lost ≥ 15% body weight at randomization (Week 60). Percent change in body weight from Week 0 to Week 112 will be conducted in a manner similar to the primary efficacy analyses. Participants who are randomized at Week 60, regardless of achievement of the body weight plateau at Week 60, will be included in the analysis of this endpoint.

Exploratory measures will be completed during the weight‐loss period at baseline (Visit 2) and at Week 60 (Visit 14) and at multiple time points throughout the weight‐maintenance period and include patient‐reported outcomes assessments using the following self‐administered questionnaires: Power of Food Scale (PFS) [32, 33], Food Craving Questionnaire‐trait‐reduced (FCQ‐T reduced) [34], and the Short Form 36 version 2 (SF‐36v2), acute, 1 week recall version‐Adult [35].

## 3 Results

The SURMOUNT‐MAINTAIN trial is ongoing and expected to be completed by early 2026. A total of 441 participants were enrolled. Table 3 presents the baseline demographic and clinical characteristics of participants. In SURMOUNT‐MAINTAIN, mean (SD) age was 47 years (13.02) with a mean body weight of 114 kg (26.97), mean BMI of 40 kg/m^2^ (8.07) and mean waist circumference of 119 cm (17.56) at baseline. The majority of participants were female (65%) and of White race (67%). Mean (SD) baseline HbA1c was 5.64% (0.36) and systolic blood pressure was 126 mmHg (12.85). Overall, 61% of participants had prediabetes, 44% had hypertension, and 33% had dyslipidemia at baseline. The majority of participants reported at least one or two obesity‐related comorbidities at baseline.

**TABLE 3:** Key baseline demographics and clinical characteristics of enrolled participants.

	Week 0 (start of 60‐week open‐label weight‐loss period with tirzepatide) (*N* = 441)
Age, years	46.6 ± 13.02
< 65 years	391 (88.7)
≥ 65 years	50 (11.3)
Sex, *n* (%)	
Male	153 (34.7)
Female	288 (65.3)
Race, *n* (%)	
White	295 (67.4)
Black or African American	107 (24.4)
Asian	20 (4.6)
Multiple	11 (2.5)
American Indian or Alaska Native	3 (0.7)
Not reported	3 (0.7)
Native Hawaiian or other Pacific Islander	2 (0.5)
Hispanic or Latino ethnic group, *n* (%)	99 (22.4)
Duration of obesity, years	15.1 ± 11.10
Body weight, kg	113.8 ± 26.97
BMI, kg/m^2^	40.1 ± 8.07
BMI category, *n* (%)	
< 30	20 (4.5)
≥ 30 to < 35	111 (25.2)
≥ 35 to < 40	122 (27.7)
≥ 40	188 (42.6)
Waist circumference, cm	119.4 ± 17.56
HbA1c, %	5.64 ± 0.36
Systolic blood pressure, mmHg	126.3 ± 12.85
Diastolic blood pressure, mmHg	81.3 ± 8.19
Pulse, bpm	71.9 ± 9.80
Lipid parameters, mg/dL	
Total cholesterol	192.0 ± 36.96
HDL cholesterol	50.6 ± 13.34
LDL cholesterol	115.6 ± 31.12
VLDL cholesterol	25.2 ± 11.77
Triglycerides	128.5 ± 70.17
Estimated GFR, mL/min/1.73 m^2^	89.7 ± 19.51
Comorbidities, *n* (%)	
Prediabetes	268 (60.8)
Impaired glucose metabolism	4 (0.9)
Hypertension	194 (44.0)
Dyslipidemia	146 (33.1)
Obstructive sleep apnea	83 (18.8)
Back pain	78 (17.7)
Reproductive system disorder	60 (13.6)
Osteoarthritis	45 (10.2)
Anxiety/depression	155 (35.1)
Nonalcoholic fatty liver disease/nonalcoholic steatohepatitis	36 (8.2)
Asthma	57 (12.9)
Gallbladder disease	55 (12.5)
Dermatologic condition	26 (5.9)
Gout/hyperuricemia	18 (4.1)
Renal disease	11 (2.5)
Urinary incontinence	11 (2.5)
Cerebrovascular disease	9 (2.0)
Venous thromboembolism	9 (2.0)
Atrial fibrillation	8 (1.8)
Coronary artery disease	7 (1.6)
Peripheral vascular disease	4 (0.9)
Heart failure	2 (0.5)
Oncologic disease	2 (0.5)
Motor dysfunction	1 (0.2)
Number of comorbidities, *n* (%)	
0	83 (18.8)
1	115 (26.1)
2	70 (15.9)
3	62 (14.1)
4	45 (10.2)
≥ 5	66 (15.0)

*Note*: Data are mean ± SD, unless otherwise stated.

Abbreviations: GFR, glomerular filtration rate; HbA1c, glycated hemoglobin; HDL, high‐density lipoprotein; LDL, low‐density lipoprotein; VLDL, very low‐density lipoprotein.

## 4 Discussion

SURMOUNT‐MAINTAIN aims to evaluate maintenance treatment strategies among individuals with obesity or overweight who experienced weight reduction with tirzepatide MTD. The maintenance treatment strategies will include: (1) reducing the dose of tirzepatide or (2) continuing tirzepatide MTD, and each strategy will be compared to switching to placebo. Although the study is not specifically powered to directly compare the two strategies, it may establish a foundation for further research in the future, potentially guiding more targeted investigations in these areas.

A clinical challenge is that intentional weight reduction, whether through lifestyle intervention or additional treatment with OMMs, eventually levels out or “plateaus.” Given that clinically meaningful body weight reduction is considered 5% or greater [36, 37] and given that 1%–2% change in body weight is considered a normal physiological fluctuation [38], it could be postulated that a less than 5% body weight change within 3 months following an initial intentional body weight reduction during the 60‐week weight‐loss period might reasonably reflect a clinical body weight plateau, which is the definition used in this study.

Although no specific cutoff for weight regain has been identified to maximize risk difference between maintenance and regain, data from the Look AHEAD study demonstrated that maintaining at least 75% of weight loss generally helped preserve cardiometabolic benefits associated with weight reduction [39]. A study in a bariatric surgery cohort found that across multiple thresholds for weight regain assessed in the study, 20% or greater weight regain correlated best with worsening cardiometabolic risk factors [40]. Based on these data, maintaining at least 75%–80% of initial body weight reduction regardless of therapeutic modality might be expected to be clinically beneficial and serves as the rationale for the threshold of 80% maintenance of body weight reduction chosen as one of the study objectives.

The primary objective of this study will evaluate the percent maintenance of body weight reduction achieved during the weight‐loss period among those who have reached a body weight plateau. Additionally, we will evaluate maintenance of at least 15% body weight reduction for participants who lost at least 15% body weight at the end of the weight‐loss period. Such magnitude of body weight reduction is possible with highly effective OMMs such as tirzepatide and is associated with metabolic benefits including diabetes prevention and improvement in cardiovascular risk factors, such as hypertension and dyslipidemia [36, 41]. In summary, several study objectives aim to evaluate maintenance of body weight reduction through continuous, categorical, and target‐based approaches in the absence of a widely accepted definition in the obesity field for successful maintenance of body weight reduction.

In this study, all patients will receive active medication, that is tirzepatide, in the open‐label weight‐loss period and will receive rescue tirzepatide (i.e., re‐initiation or increased dose) if there is excess weight regain (defined as 50% or more weight regain) during the weight‐maintenance period. Unfortunately, many people living with obesity (and their clinicians) are under the misunderstanding that OMMs can be stopped after achieving initial body weight reduction, which is a misperception in treatment approach and a bias not applied to other chronic diseases. For example, it is mostly understood that discontinuing medication treatment of hypertension or dyslipidemia after reaching therapeutic goals will lead to loss of improvement in blood pressure and blood lipids, which is a straightforward concept that is not always appreciated in medication treatment of obesity. The rescue tirzepatide approach is designed to limit both the physiological and psychological harm of weight regain given the anticipated weight regain with stopping or reducing the dose of OMMs.

In addition to SURMOUNT‐MAINTAIN, future studies are warranted to investigate multiple strategies targeting different medications and medical interventions, as well as behavioral and environmental factors to help achieve maintenance of body weight reduction. For example, it will be important to understand if some people with obesity could succeed in maintenance of body weight reduction by switching to a different OMM for maintenance of weight reduction (e.g., ATTAIN‐MAINTAIN Study NCT06584916 [42]). Additionally, it remains to be understood whether the method and magnitude of body weight reduction play a role in the ability to maintain the reduced body weight and what the predictors are for successful maintenance of body weight reduction and associated cardiometabolic benefits. As the number of available highly effective OMMs is expected to increase in the future, it will become increasingly more important to understand which combinations of OMMs and lifestyle interventions are best suited to initiate body weight reduction versus maintain body weight reduction and how best to individualize the use of OMMs.

## 5 Conclusion

The SURMOUNT‐MAINTAIN study aims to evaluate maintenance treatment strategies among adults with obesity and overweight who experienced weight reduction with the MTD of tirzepatide. These maintenance treatment strategies will include: (1) reducing the dose of tirzepatide and (2) continuing the MTD dose of tirzepatide; and each strategy will be compared to switching to placebo. This study will additionally evaluate the degree of maintenance of body weight reduction as continuous, categorical, and target‐based parameters, explore in parallel changes in cardiometabolic parameters, and generate data on the use of rescue OMMs in case of weight regain above a predefined threshold. SURMOUNT‐MAINTAIN may provide clinicians with evidence for additional options to support patient‐centered strategies for the maintenance of body weight reduction in adults living with obesity.

## Conflicts of Interest

D.B.H. has acted as a consultant, advisory board member, and speaker for Eli Lilly and Company and Novo Nordisk; has acted as an advisory board member for Amgen; has served as a consultant for AstraZeneca and Zealand; and has received institutional research funding from Eli Lilly and Company, KVK Tech, Novo Nordisk, and Weight Watchers. L.J.A. reports receiving grants or personal fees from Altimmune, AstraZeneca, Boehringer Ingelheim, Eli Lilly and Company, ERX, Gelesis, Intellihealth, Jamieson Wellness, Janssen, Novo Nordisk, Optum, Pfizer, Senda Biosciences, and Versanis and being a shareholder of ERX Pharmaceuticals, Gelesis, Intellihealth, and Jamieson Wellness. S.W. reported receiving nonfinancial support from Eli Lilly and Company during the conduct of the study and personal fees from Novo Nordisk, Boehringer Ingelheim, Biohaven, Bausch Health Canada, and Eli Lilly and Company outside the submitted work. H.E.B.'s research site institution has received research grants from 89Bio, Alon Medtech/Epitomee, Altimmune, Amgen, AstraZeneca, Bioage, Bionime, Boehringer Ingelheim, Carmot, Chorus/Bioage, Eli Lilly and Company, GlaxoSmithKline, Novartis, Novo Nordisk, Pfizer, Regeneron, Satsuma, Selecta, Shionogi, Skye/Birdrock, Veru, Viking, Vivus, and Zomagen; and he has served as a consultant for 89Bio, Altimmune, Amgen, Boehringer Ingelheim, Eli Lilly and Company, Kiniksa, Novo Nordisk, Regeneron, Rivus, Veru, Zomagen, and ZyVersa. E.G.‐V., A.D.A., P.S., J.P.D., C.S., and C.J.L. are employees and shareholders of Eli Lilly and Company.

## Supporting information


**Data S1.** oby70014‐sup‐0001‐supinfo.

## Data Availability

Lilly provides access to all individual participant data collected during the trial, after anonymization, with the exception of pharmacokinetic or genetic data. Data are available to request 6 months after the indication studied has been approved in the US and EU and after primary publication acceptance, whichever is later. No expiration date for data requests is currently set once data are made available. Access is provided after a proposal has been approved by an independent review committee identified for this purpose and after receipt of a signed data sharing agreement. Data and documents, including the study protocol, statistical analysis plan, clinical study report, and blank or annotated case report forms, will be provided in a secure data sharing environment. For details on submitting a request, see the instructions provided at www.vivli.org.
