# Supplementary material for: Tirzepatide for the Maintenance of Body Weight Reduction: Rationale, Design, and Baseline Characteristics of SURMOUNT‐MAINTAIN
Source: Obesity (Silver Spring). 2025 Sep 7;33(10):1873–85. doi: 10.1002/oby.70014 (PMC12477106; doi:10.1002/oby.70014)
Supplement: Supplementary file 1 — Data S1. oby70014‐sup‐0001‐supinfo. [file OBY-33-1873-s001.docx]

**SUPPLEMENTARY APPENDIX**

**CONTENTS**

**SUPPLEMENTAL METHODS**

INCLUSION AND EXCLUSION CRITERIA............................................................................... 2

DISCONTINUATION OF STUDY INTERVENTION................................................................. 6

PARTICIPANT DISCONTINUATION/WITHDRAWAL FROM THE STUDY......................... 8

LOST TO FOLLOW-UP................................................................................................................. 8

PRIMARY ESTIMAND................................................................................................................. 8

SECONDARY ESTIMAND........................................................................................................... 9

**ELIGIBILITY CRITERIA**

**Inclusion Criteria**

Participants are eligible to be included in the study only if all of the following criteria apply:

***Age***

1. Participants must be 18 years of age or of an acceptable age to provide informed consent according to local regulations, whichever is older.

***Weight and type of participant and disease characteristics***

1. Have a BMI of

- ≥30 kg/m^2^ or
- ≥27 kg/m^2^ and previously diagnosed with at least 1 of the following weight-related comorbidities:
  - hypertension: treated or with systolic blood pressure ≥130 mmHg or diastolic blood pressure ≥80 mmHg
  - dyslipidemia: treated or with low-density lipoprotein (LDL) ≥160 mg/dL (4.1 mmol/L) or triglycerides ≥150 mg/dL (1.7 mmol/L), or high-density lipoprotein (HDL) <40 mg/dL (1.0 mmol/L) for men or <50 mg/dL (1.3 mmol/L) for women
  - obstructive sleep apnea
  - cardiovascular disease, for example, ischemic cardiovascular disease, New York Heart Association Functional Classification Class I-III heart failure.

1. Have a history of at least 1 self-reported unsuccessful dietary effort to lose BW
2. In the investigator’s opinion, are well motivated, capable, and willing to

- learn how to self-inject study intervention, as required for this protocol (visually impaired persons who are not able to perform the injections must have the assistance of a sighted individual trained to inject the study intervention; persons with physical limitations who are not able to perform the injections must have the assistance of an individual trained to inject the study intervention)
- inject study intervention (or receive an injection from a trained individual if visually impaired or with physical limitations), and
- follow study procedures for the duration of the study, including, but not limited to, following lifestyle advice (for example, dietary restrictions and exercise plan), maintaining a study diary, and completing required questionnaires
  - for visually impaired participants, they must have the assistance of a sighted individual for maintaining 3-day diet and exercise log to be completed prior to each counseling visit

***Sex and contraceptive/barrier requirements***

Contraceptive use by participants should be consistent with local regulations regarding the methods of contraception for those participating in clinical studies.

1. Male and female participants: for the contraception requirements of this protocol, see Section 10.4, Appendix 4

***Informed consent***

1. Are capable of giving signed informed consent as described in Appendix 1, which includes compliance with the requirements and restrictions listed in the informed consent form (ICF) and in this protocol

**Exclusion Criteria**

Participants are excluded from the study if any of the following criteria applies:

***Medical conditions***

***Diabetes related***

1. Have type 1 diabetes (T1D) or T2D, a history of ketoacidosis, or hyperosmolar state or

coma

1. Have at least 1 laboratory value suggestive of diabetes during screening, including 1 or more of hemoglobin A1c (HbA1c) ≥6.5% (≥48 mmol/mol), fasting glucose ≥126 mg/dL (≥7.0 mmol/L), or random glucose ≥200 mg/dL (≥11.1 mmol/L)

***Obesity related***

1. Have a self-reported change in BW >5 kg within 3 months prior to screening
2. Have a prior or planned surgical treatment for obesity, excluding liposuction or abdominoplasty if performed >1 year prior to screening
3. Have or plan to have endoscopic and/or device-based therapy for obesity or have had device removal within the last 6 months prior to screening.

Examples:

- mucosal ablation
- gastric artery embolization
- intragastric balloon, and
- duodenal-jejunal endoluminal liner

***Other medical***

1. Have renal impairment measured as estimated glomerular filtration rate (eGFR) <30 mL/min/1.73 m^2^, calculated by Chronic Kidney Disease Epidemiology as determined by central laboratory during screening
2. Have a known clinically significant gastric emptying abnormality, for example, severe gastroparesis or gastric outlet obstruction, or chronically take drugs that directly affect GI motility
3. Have a history of chronic or acute pancreatitis
4. Have evidence of a significant uncontrolled endocrine abnormality, for example, thyrotoxicosis or adrenal crises, in the opinion of the investigator

*Note: Participants receiving treatment for hypothyroidism may be included, provided their thyroid hormone replacement dose has been stable for at least 3 months*

*Note: Participants with a history of subclinical hypothyroidism may be included if, in the investigator’s opinion, the participant is unlikely to require initiation of thyroid hormone replacement during the course of the study*

1. Have obesity induced by other endocrinologic disorders, for example, Cushing syndrome, or diagnosed monogenetic or syndromic forms of obesity, for example, Melanocortin 4 Receptor deficiency or Prader Willi Syndrome
2. Have a history of significant active or unstable Major Depressive Disorder or other severe psychiatric disorder, for example, schizophrenia, bipolar disorder, or other serious mood or anxiety disorder, within the last 2 years

*Note: Participants with Major Depressive Disorder or generalized anxiety disorder whose disease state is considered stable for the past 2 years and expected to remain stable throughout the course of the study, in the opinion of the investigator, may be considered for inclusion if they are not on excluded medications*

1. Have a Patient Health Questionnaire-9 (PHQ-9) score of 15 or more on or before Visit 2
2. Are in the opinion of the investigator, actively suicidal or have any lifetime history of a suicide attempt
3. On the Columbia-Suicide Severity Rating Scale (C-SSRS) on or before Visit 2

- have answered “yes” to either Question 4 or Question 5 on the “Suicidal Ideation” portion of the C-SSRS or
- have answered “yes” to any of the suicide-related behaviors on the “Suicidal Behavior” portion of the C-SSRS, **and**
- the ideation or behavior occurred within the past month.

1. Have uncontrolled hypertension (systolic blood pressure above or equal to 160 mmHg and/or diastolic blood pressure above or equal to 100 mmHg)
2. Have any of the following cardiovascular conditions within 3 months prior to Visit 2

- acute myocardial infarction
- cerebrovascular accident (stroke)
- unstable angina, and
- hospitalization due to congestive heart failure (CHF)

1. Have New York Heart Association Functional Classification Class IV CHF
2. Have acute or chronic hepatitis, signs and symptoms of any other liver disease other than nonalcoholic fatty liver disease, or any of the following, as determined by the central laboratory during screening

- alanine aminotransferase (ALT) level >3.0x upper level of normal (ULN) for the reference range
- alkaline phosphatase (ALP) level >1.5x ULN for the reference range, or
- total bilirubin (TBL) level >1.5x ULN for the reference range, except for cases of known Gilbert’s Syndrome

*Note: Participants with nonalcoholic fatty liver disease (NAFLD) are eligible to participate in this trial if their ALT level is ≤3.0x ULN for the reference range*

1. Have a serum calcitonin level (at Visit 1) of

- ≥20 ng/L, if eGFR ≥60 mL/min/1.73 m^2^, and
- ≥35 ng/L, if eGFR <60 mL/min/1.73 m^2^

1. Have a family or personal history of medullary thyroid cancer (MTC) or multiple endocrine neoplasia syndrome type 2
2. Have a history of an active or untreated malignancy or are in remission from a clinically significant malignancy (other than basal or squamous cell skin cancer, in situ carcinomas of the cervix, or in situ prostate cancer) for less than 5 years
3. Have any other condition not listed in this section, for example, hypersensitivity or intolerance, that is a contraindication to GLP-1 RAs or GIP/GLP-1 RA
4. Have a history of any other condition, such as known drug or alcohol abuse, diagnosed eating disorder, or other psychiatric disorder, that, in the opinion of the investigator, may preclude the participant from following and completing the protocol
5. Have a history of use of marijuana or tetrahydrocannabinol-containing products within 3 months of enrollment or unwillingness to abstain from marijuana or tetrahydrocannabinol-containing products use during the trial

*Note: If a participant has used cannabidiol oil during the past 3 months but agrees to refrain from use for the duration of the study, the participant can be enrolled.*

1. Have had a transplanted organ (corneal transplants [keratoplasty] allowed) or awaiting an organ transplant
2. Have any hematological condition that may interfere with HbA1c measurement, for example, hemolytic anemias and sickle cell disease

***Prior/concomitant therapy***

1. Have used a GLP-1 RA (prescribed or in a clinical study) within 12 months of screening

Examples include, but are not limited to

- Saxenda® (liraglutide injection 3.0 mg)
- Wegovy® (semaglutide injection 2.4 mg)

1. Are receiving or have received within 3 months prior to screening chronic (>2 weeks or >14 days) systemic glucocorticoid therapy (excluding topical, intraocular, intranasal, intra-articular, or inhaled preparations) or have evidence of a significant, active autoimmune abnormality (for example, lupus or rheumatoid arthritis) that has required (within the last 3 months) or is likely to require, in the opinion of the investigator, concurrent treatment with systemic glucocorticoids (excluding topical, intraocular, intranasal, intra-articular, or inhaled preparations) during the course of the study
2. Have current or a history of (within 3 months of screening) treatment with medications that may cause significant weight gain, including but not limited to, tricyclic antidepressants, atypical antipsychotics, and mood stabilizers

Examples:

- imipramine
- amitriptyline
- mirtazapine
- paroxetine
- phenelzine
- chlorpromazine
- thioridazine
- clozapine
- olanzapine
- valproic acid (and its derivatives), and
- lithium

*Note: Selective serotonin reuptake inhibitors other than paroxetine are permitted.*

1. Have taken, within 3 months prior to screening, any non-GLP-1 RA medications (prescribed or over-the-counter) or alternative remedies that promote weight loss. Examples include, but are not limited to:

- Adipex® (phentermine)
- Belviq® (lorcaserin)
- Bontril® (phendimetrazine)
- Qsymia® (phentermine/topiramate combination)
- Contrave® (naltrexone/bupropion)
- Plenity® (oral superabsorbent hydrogel)
- Xenical®/Alli® (orlistat)
- Meridia (sibutramine)
- Acutrim (phenylpropanolamine), and
- Sanorex (mazindol)

*Note: Use of metformin or any other glucose-lowering medication, whether prescribed for polycystic ovary syndrome or diabetes prevention, is not permitted.*

***Prior/concurrent clinical study experience***

1. Are currently enrolled in any other clinical study involving an investigational product or any other type of medical research judged not to be scientifically or medically compatible with this study
2. Within the last 30 days of Visit 2, have participated in a clinical study and received treatment, whether active or placebo. If the study involved an IP, 5 half-lives or 30 days, whichever is longer, should have passed.
3. Have ever taken tirzepatide (prescribed) or previously completed or withdrawn from this study or any other study investigating tirzepatide after receiving at least 1 dose

***Other exclusion criteria***

1. Are investigator site personnel directly affiliated with this study and/or their immediate families. Immediate family is defined as a spouse, parent, child, or sibling, whether biological, or legally adopted
2. Are Eli Lilly and Company employees.

**DISCONTINUATION OF STUDY INTERVENTION**

When necessary, a participant may be permanently discontinued from study intervention. If study intervention is permanently discontinued during the open-label Weight-Loss Period, the participant will not be randomized and will be permanently discontinued from study. If study intervention is permanently discontinued after randomization, the participant will remain in the study to complete all procedures and visits as shown in the study protocol Schedule of Activities. A participant should be permanently discontinued from study intervention if

- **participant decision**
  - the participant requests to discontinue study intervention
- **clinical considerations**
  - initiation of GLP-1 RA, DPP-4 inhibitor, or GIP/GLP-1 RA obtained outside the study if participants will not, or cannot, discontinue them
  - initiation of additional approved prescription OMM, if participants will not, or cannot discontinue them
  - has bariatric surgery or weight-loss procedure or any female participant who becomes pregnant while participating in the study
  - BMI of ≤18.5 kg/m^2^ is reached at any time during the treatment period

Note: The investigator should contact the sponsor CRP to discuss whether it is medically appropriate for the participant to continue study treatment.

- - significant GI symptoms despite management

Note: The investigator should contact the sponsor CRP to discuss whether it is medically appropriate for the participant to continue study treatment

- - significant elevation of calcitonin
  - occurrence of any other TEAE, SAE, or clinically significant finding for which the investigator believes that permanent study intervention discontinuation is the appropriate measure to be taken
  - diagnosis of
    - T1D
    - thyroid C-cell hyperplasia, metastatic thyroid cancer (MTC), or multiple endocrine neoplasia type 2 (MEN-2) after randomization, and
    - acute or chronic pancreatitis
  - an active or untreated malignancy (other than basal or squamous cell skin cancer, in situ carcinomas of the cervix, or in situ prostate cancer) after randomization
  - in the opinion of the investigator, the participant should permanently discontinue the study intervention for safety reasons
- **• Suicidal ideation and behavior**
  - PHQ-9 score ≥15

Participants should be referred to a mental health professional to assist in deciding whether the participant should be discontinued from study intervention. If a participant’s psychiatric disorder can be adequately treated with psycho- and/or pharmacotherapy, then the participant, at the discretion of the Investigator (in agreement with the mental health professional), may be continued in the trial on randomized therapy

- - Study intervention may be discontinued if participants
    - answered “yes” to Question 4 or Question 5 on the “Suicidal Ideation” portion of the C-SSRS, or
    - answered “yes” to any of the suicide-related behaviors on the Suicidal Behavior portion of the C-SSRS

A psychiatrist or appropriately trained professional may assist in the decision to discontinue the participant.

- **Hypersensitivity reactions**
  - If the investigator determines that a systemic hypersensitivity reaction has occurred related to study intervention administration, the participant may be permanently discontinued from the study intervention, and the sponsor’s designated medical monitor should be notified. If the investigator is uncertain about whether a systemic hypersensitivity reaction has occurred and whether discontinuation of study intervention is warranted, the investigator may consult the sponsor.

**PARTICIPANT DISCONTINUATION AND STUDY WITHDRAWAL**

Discontinuation is expected to be uncommon. A participant may withdraw from the study at any time

- at the participant’s own request for any reason or without providing any reason
- at the discretion of the investigator for safety, behavioral, compliance, or administrative reasons
- if enrolled in any other clinical study involving an investigational product, or enrolled in any other type of medical research judged not to be scientifically or medically compatible with this study

A participant will withdraw from the study

- if a female participant becomes pregnant, or
- if participant undergoes bariatric surgery or a weight-loss procedure, for example, gastric balloon placement, during the study

Participation in the study can be stopped for medical, safety, regulatory, or other reasons consistent with applicable laws, regulations, and good clinical practice. Participants who agree to provide information relevant to any trial endpoint at the end of the study are not considered to have discontinued from the study.

At the time of discontinuing from the study, if possible, the participant will complete procedures for an early discontinuation visit, if applicable, as shown in the Schedule of Activities. If the participant has not already discontinued the study intervention, the participant will be permanently discontinued from the study intervention at the time of the decision to discontinue the study.

If the participant withdraws consent for disclosure of future information, the sponsor may retain and continue to use any data collected before such a withdrawal of consent. If a participant withdraws from the study, the participant may request destruction of any samples taken and not tested, and the investigator must document this in the site study records.

**LOST TO FOLLOW-UP**

A participant will be considered lost to follow-up if they repeatedly fail to return for scheduled visits and are unable to be contacted by the study site. Site personnel or designee are expected to make diligent attempts to contact participants who fail to return for a scheduled visit or were otherwise unable to be followed up by the site.

**PRIMARY ESTIMAND**

The primary estimand evaluated in this study is the efficacy estimand. This estimand focuses on the treatment effect if participants who underwent randomization continued to receive the study treatment without taking other OMMs, GLP-1 RAs, GIP/GLP-1 RA, or DPP-4 inhibitors, bariatric surgery or weight-loss procedures, or rescue tirzepatide. The primary estimand for this study aims to answer the following question:

- what is the treatment difference between tirzepatide 5 mg and/or MTD vs placebo in mean percent maintenance of body weight reduction assuming that participants had stayed on treatment, had not taken prohibited therapy, and assuming that participants who took rescue tirzepatide would not have received any additional improvement from their randomized study treatment?

**SECONDARY ESTIMAND**

The secondary estimand evaluated in this study is a modified treatment-regimen estimand. This estimand aims at reflecting how participants with obesity or overweight with at least 1 weight-related comorbid condition are treated in clinical practice and takes into account both tolerability and efficacy. This modified treatment-regimen estimand answers the following question of interest for the primary objective:

- What is the treatment difference between tirzepatide 5 mg and/or MTD and placebo in mean percent maintenance of body weight reduction achieved during the 60-week Weight-Loss Period at Week 112 regardless of treatment discontinuation, initiation of prohibited therapy, and assuming that participants who had bariatric surgery or another weight-loss procedure or took rescue tirzepatide would not have received any additional improvement from their randomized study treatment
